# Standardised data reporting from pre-hospital advanced airway management – a nominal group technique update of the Utstein-style airway template

**DOI:** 10.1186/s13049-018-0509-y

**Published:** 2018-06-04

**Authors:** G. A. Sunde, A. Kottmann, J. K. Heltne, M. Sandberg, M. Gellerfors, A. Krüger, D. Lockey, S. J. M. Sollid

**Affiliations:** 1 Norwegian Air Ambulance Foundation, Drøbak, Norway; 2 Dept. of Anaesthesia and Intensive Care, Haukeland University Hospital, Bergen, Norway; 3 Faculty of Health Sciences, University of Stavanger, Stavanger, Norway; 4 Emergency Dept., University Hospital of Lausanne, Lausanne, Switzerland; 5 Swiss Air Ambulance – Rega, Zürich, Switzerland; 6 Dept. of Medical Sciences, University of Bergen, Bergen, Norway; 7 Air Ambulance Dept., Oslo University Hospital, Oslo, Norway; 8 Faculty of Medicine, University of Oslo, Oslo, Norway; 9 Karolinska Institutet, Dept. of Clinical Science and Education, Section of Anaesthesiology and Intensive Care, Stockholm, Sweden; 10 Swedish Air Ambulance (SLA), Mora, Sweden; 11 Dept. of Anaesthesiology and Intensive Care, Södersjukhuset, Stockholm, Sweden; 12 Dept. of Emergency Medicine and Pre-hospital Services, St. Olavs Hospital, Trondheim, Norway; 13 London’s Air Ambulance, Bartshealth NHS Trust, London, UK

**Keywords:** Airway management, Air ambulances, Emergency medical services, Intubation, Data accuracy

## Abstract

**Background:**

Pre-hospital advanced airway management with oxygenation and ventilation may be vital for managing critically ill or injured patients. To improve pre-hospital critical care and develop evidence-based guidelines, research on standardised high-quality data is important. We aimed to identify which airway data were most important to report today and to revise and update a previously reported Utstein-style airway management dataset.

**Methods:**

We recruited sixteen international experts in pre-hospital airway management from Australia, United States of America, and Europe. We used a five-step modified nominal group technique to revise the dataset, and clinical study results from the original template were used to guide the process.

**Results:**

The experts agreed on a key dataset of thirty-two operational variables with six additional system variables, organised in time, patient, airway management and system sections. Of the original variables, one remained unchanged, while nineteen were modified in name, category, definition or value. Sixteen new variables were added. The updated dataset covers risk factors for difficult intubation, checklist and standard operating procedure use, pre-oxygenation strategies, the use of drugs in airway management, airway currency training, developments in airway devices, airway management strategies, and patient safety issues not previously described.

**Conclusions:**

Using a modified nominal group technique with international airway management experts, we have updated the Utstein-style dataset to report standardised data from pre-hospital advanced airway management. The dataset enables future airway management research to produce comparable high-quality data across emergency medical systems. We believe this approach will promote research and improve treatment strategies and outcomes for patients receiving pre-hospital advanced airway management.

**Trial registration:**

The Regional Committee for Medical and Health Research Ethics in Western Norway exempted this study from ethical review (Reference: REK-Vest/2017/260).

**Electronic supplementary material:**

The online version of this article (10.1186/s13049-018-0509-y) contains supplementary material, which is available to authorized users.

## Background

Pre-hospital advanced airway management (PHAAM) with the control of oxygenation and ventilation is vital in the management of critically ill or injured patients in the field and may contribute to better outcomes [1–3]. Results from research on PHAAM are challenged by heterogeneity in provider competence, airway techniques, and the quality of data collected in many airway studies [4]. To improve pre-hospital critical care and to develop evidence-based guidelines, research based on standardised high-quality data is important [5, 6]. Using a common and uniform set of data definitions may be the first step in such a process [7].

In pre-hospital critical care research, there has been an acceptance and tradition for using structured consensus methods to evaluate interventions, to develop guidelines, and for educational and research purposes [8, 9]. Templates for documenting and reporting of standardised data have been developed by similar methodology for out-of-hospital cardiac arrest, paediatric advanced life support, in-hospital cardiac arrest resuscitation, major incidents and disaster management, laboratory cardiopulmonary research, major trauma, emergency medical dispatch, physician staffed emergency medical services and drownings [10–19]. Developments in airway management devices, airway management strategies and training, along with patient safety issues; require that such templates are updated on a regular basis like clinical guidelines and recommendations [20].

An Utstein-style airway template was published in 2009 by an international airway expert group [21]. The feasibility of collecting standardised airway data across different patient populations and international emergency medical services (EMS) have been described [22]. The aim of this study was to update and revise the Utstein-style template for the reporting of PHAAM data, using a nominal group technique (mNGT) with international experts to identify which data variables would be most important to document today.

## Methods

### Study design

The revision of the Utstein-style airway template was performed using a modified nominal group technique (mNGT) consensus process, which has previously proven useful in the development of templates and guidelines for pre-hospital critical care [9, 14].

### Pre-hospital advanced airway management

In the original template, advanced airway management was defined as the attempted insertion of an advanced airway adjunct or the administration of ventilatory assistance, in this context being “*any airway management beyond manual opening of the airway and the use of simple adjuncts, such as an* oropharyngeal airway”. This type of management includes the use of a supraglottic airway device (SAD), tracheal intubation (TI), or emergency front of neck access (eFONA).

### Data variable

A data variable should be clearly defined to avoid misinterpretation. Data points should be simple to register and possible to integrate into existing registries [21]. This requires a data variable dictionary containing information on data number, name, type of data, categories or values and definition of data variable [14].

### Group of experts

The recruited experts were clinicians with leadership experience from pre-hospital critical care, had made substantial contributions to airway management research or airway management guidelines, or were considered experts in the field of PHAAM. They were recruited from networks such as European Pre-hospital Research Alliance (EUPHOREA) and the European Airway Management Society (EAMS). The experts were invited by individual email and were not aware of the composition of the group until the final consensus meeting.

### The modified nominal group process

The mNGT is a systematic qualitative method involving questionnaires in repeated rounds with a final meeting aimed at consensus [9]. Our mNGT included three email rounds with questionnaires and answers (QA), and a one-day consensus meeting for plenary discussions. A fourth email round was included after the meeting for minor adjustments or comments. The results from each round were used to guide the development of the questionnaires for the following round. A third party distributed and managed the responses from the experts in individual emails and anonymised the answers. The mNGT was run from February to August 2017. The final dataset was forwarded to the experts for approval.

### First email round

An Excel spreadsheet (Microsoft Corporation, Redmond, WA, USA) with the original template variables was sent to the experts (Additional file 1: Table S1). The experts were instructed to rate each variable on a 5-point Likert scale (from 1 = “totally disagree” to 5 = “totally agree”) according to how important the variable was considered to be for PHAAM and to indicate whether the original variable should be changed. The experts were then requested to suggest between three and five new variables. Additional free-text comments were allowed. These comments were not distributed to the other experts but were used to revise the variables.

### Second email round

The revised variables were organised in the original template sections, with the suggested new variables in the “optional variables section” (Additional file 2: Table S2). The experts were instructed to rank the most important variables within each section. Where relevant and for the ranked variables only, the experts indicated whether changes were warranted. Additional free-text comments were also possible in this round.

### Third email round

The instructions for ranking and suggestions were similar to those of the previous round, with the revised variables now grouped in core-system, core-patient, core-post-intervention, or fixed-system sections (Additional file 3: Table S3).

### The consensus meeting

The aim of the consensus meeting was to finalise the variable set and discuss items that had not been cleared during the first three rounds. The main results after the email rounds were presented, and the expert comments from the preceding rounds were used to guide the discussions. The experts agreed by consensus on the changes to the template structure or variables.

### Ranking

We measured expert commitment towards each variable as the number of times the individual variable was nominated by the experts. Within each section, the variables rated as “most important” received the highest score, and those rated as “least important” received the lowest score. If two variables scored equally, the variable with the highest number of individual nominations, compared to those with highest rating, was ranked higher.

## Results

### Experts

Twenty-one experts were invited to join the mNGT-process, of whom sixteen participated in all email rounds. The experts were recruited from Australia, United States of America, and Europe. The level and type of airway experience, along with country of origin of the expert group, is described in Additional file 4. Of the experts who participated, eight attended the final consensus meeting, along with five members of the project steering group.

### Definition of PHAAM

The expert group decided to keep the definition of advanced airway management unchanged from the original template, as **“*the attempted insertion of an advanced airway adjunct or administration of ventilatory assistance***”.

### First email round

**Fig. 1:**
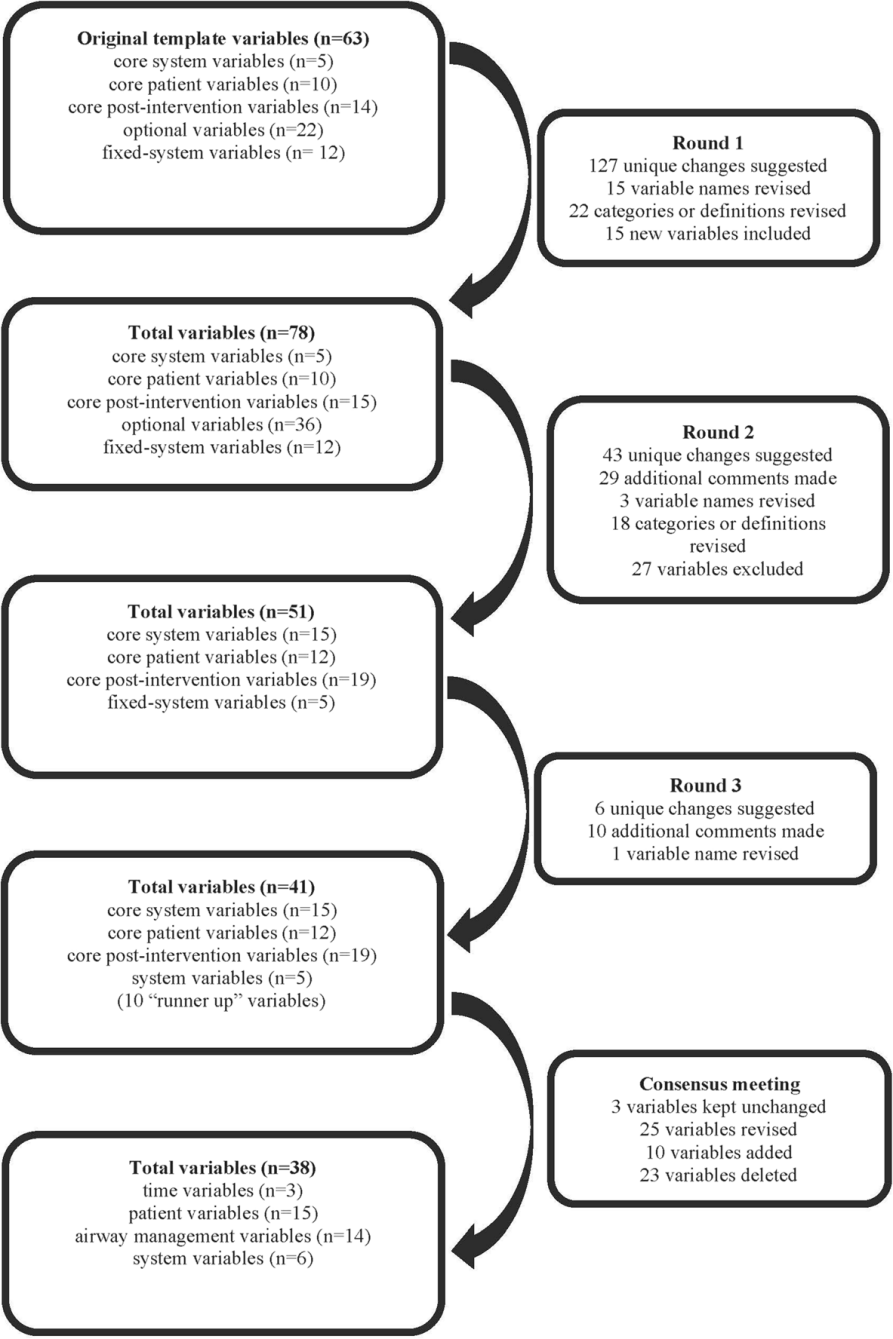
Flowchart describing the modified nominal group technique. The original variables were modified or deleted, and new variables inserted according to the experts’ comments and suggestions at each stage. Similar suggestions and variables were merged

The experts made 127 unique suggestions for changes in variable names, categories or values in 28 (44%) of the original variables. After merging similar suggestions and variables, 15 variable names and 22 categories were revised. Fifteen new variables were added to the dataset before the second email round (Additional file 2: Table S2). This process is detailed in the flowchart (Fig. 1).

### Second email round

The experts indicated that change was warranted for 24 (18%) variables and suggested 43 unique changes. Twenty-nine additional comments to improve the variables were submitted. After ranking and merging similar suggestions, 27 variables were cut, leaving 51 variables to be included in email round three. Following the experts’ suggestions, the optional section was removed, and its variables were distributed in the remaining sections (Additional file 3: Table S3).

### Third email round

In this round, the experts indicated a need for change in the variable name, category or definition for 23 (45%) variables, and provided ten additional comments to improve the variables. The remaining variables were revised and formed the starting point for the discussions in the consensus meeting (Additional file 5: Table S4).

### The consensus meeting

The experts discussed the remaining 41 variables and 10 “runner-up” variables. The experts agreed on a dataset including 32 operational variables with an additional six system variables that were identical across all missions, compared to 63 variables in the original template (Tables 1, 2, 3, 4). Of the original variables, only one variable (age) remained unchanged. Nineteen original variables were modified in terms of the variable name, category, definition or value, and the experts added 16 new variables to the revised dataset. The experts agreed on a new template structure with time, patient, airway management and system sections. The recording of PHAAM data was to cover the interval from the patient encounter on scene to when post-intervention ventilation was established, and survival to hospital (short-term survival).

**Table 1:** Final time variables

FINAL TIME VARIABLES (data provided by provider performing the intervention)				
Number	Data variable name	Type of data	Data variable categories or values	Definition of data variable
1	Response time	Continuous	Minutes	Time from the Emergency Medical Dispatch (EMD) initiated transmission of message to the EMS unit, until the time of arrival of the EMS unit at the patient.
2	On-scene time	Continuous	Minutes	Time from EMS unit arrival at the patient until time of patient leaving scene (or time of death if dead on scene).
3	Transport time	Continuous	Minutes	Time from patient departure from scene until patient arrival at hospital.

**Table 2:** Final patient variables

FINAL PATIENT VARIABLES (data provided by provider performing the intervention)				
Number	Data variable name	Type of data	Data variable categories or values Choose only one option unless otherwise stated.	Definition of data variable
4	Age	Continuous	YY	Years rounded down. Ages under 1 year are reported in decimals (e.g. 6 months = 0.5 year)
5	Gender	Nominal	1 = Female 2 = Male 3 = Other / Unknown	Patient gender
6	Patient category	Nominal	1 = Trauma - Blunt 2 = Trauma – Penetrating 3 = Trauma - Head injury (including TBI) 4 = Trauma - Other (including burns, strangulation, drowning, or asphyxiation) 5 = Medical - Cardiac arrest 6 = Medical - Respiratory distress or breathing difficulties 7 = Medical – Intoxication 8 = Medical - Infection (including sepsis) 9 = Medical - Other (e.g. endocrinology or other medical emergencies) 10 = Neurology - Stroke (including cerebral haemorrhage or infarction) 11 = Neurology - Other (excluding stroke) 12 = Psychiatry (e.g. agitation/psychosis) 13 = Obstetrics 14 = Other emergencies, describe: ______ 15 = Unknown	Dominating reason for emergency treatment TBI = Traumatic Brain Injury
7	Indication for airway intervention	Nominal	1 = Decreased level of consciousness 2 = Hypoxemia 3 = Ineffective ventilation 4 = Existing airway obstruction 5 = Impending airway obstruction 6 = Combative or uncooperative 7 = Humanitarian (e.g. relief of pain or distress) 8 = Cardiac arrest 9 = Pre-existing airway device (e.g. SAD) not working adequately 10 = Other, describe: ____________	Indications for airway intervention. Select all that apply.
8	Patient risk factors for difficult intubation	Nominal	1 = No risk factors for difficult intubation 2 = Prior difficult intubation 3 = Reduced neck mobility, neck-immobilization device or manual in-line stabilisation (MILS) 4 = Severe obesity or thick/short neck 5 = Limited mouth opening or inter incisor distance < 4 cm 6 = Short Thyroid-Mental-Distance (< 6.5 cm) 7 = Significant maxillofacial or upper airway trauma 8 = Blood, vomit, mucus or hypersalivation in airways 9 = Pre-existing airway device (e.g. SAD) not working adequately 10 = Other, describe: ______ 11 = Risk factors not assessed.	Airway assessment before or during intervention showing patient risk factors for difficult intubation, e.g. poor visualisation, foreign body, blood or saliva. SAD = Supraglottic airway device Select all that apply.
9	Aggravating conditions for airway management	Nominal	1 = Patient entrapped during airway management 2 = Not 360-degree access to patient during airway management 3 = Suboptimal provider positioning 4 = Bright light/sunlight 5 = Darkness 6 = Hostile environment 7 = In moving helicopter/ambulance 8 = In stationary helicopter/ambulance 9 = Other, describe: ____	Patient entrapped means physically restrained in wreckage, etc. Not 360-degree access means restricted access for providers to all parts of patient, e.g. cannot move freely around patient or patient cannot be positioned on half-high stretcher for intubation. Suboptimal provider positioning means suboptimal intubating positioning, e.g. patient flat on ground during CPR with provider kneeling low or lying. Hostile environment means environment containing physical, chemical, biological, radioactive or other threats to provider safety (e.g. “active shooter” scenario). Select all that apply.
10	Respiratory rate, initial	Continuous and Nominal	1 = Number, describe 2 = NA: Did not measure 3 = NA: Could not measure	Initial value (Baseline) recorded on scene. NA = Not available
11	Blood pressure, initial	Continuous and Nominal	1 = Number, describe (Syst-BP/Dias-BP (MAP)) 2 = NA: Did not measure 3 = NA: Could not measure	Initial value (Baseline) recorded on scene. NA = Not available
12	SpO_2_, initial	Continuous and Nominal	1 = Number, describe 2 = NA: Did not measure 3 = NA: Could not measure	Initial value (Baseline) recorded on scene. NA = Not available
13	Blood pressure, lowest prior to airway management	Continuous and Nominal	1 = Number, describe (Syst-BP/Dias-BP (MAP)) 2 = NA: Did not measure 3 = NA: Could not measure	Lowest value prior to airway management recorded on scene. NA = Not available
14	SpO_2_, lowest prior to airway management	Continuous and Nominal	1 = Number, describe 2 = NA: Did not measure 3 = NA: Could not measure	Lowest value prior to airway management recorded on scene. NA = Not available
15	Blood pressure, lowest during airway management	Continuous and Nominal	1 = Number, describe (Syst-BP/Dias-BP (MAP)) 2 = NA: Did not measure 3 = NA: Could not measure	Lowest value during airway management recorded on scene. NA = Not available
16	SpO_2_, lowest during airway management	Continuous and Nominal	1 = Number, describe 2 = NA: Did not measure 3 = NA: Could not measure	Lowest value during airway management recorded on scene. NA = Not available
17	Glasgow Coma Score (GCS), initial	Continuous and Nominal	1 = Sum GCS (Motor + Verbal + Eyes) 2 = NA	Initial value (Baseline) recorded on scene. NA = Not available
18	Glasgow Coma Score (GCS), lowest prior to airway management	Continuous and Nominal	1 = Sum GCS (Motor + Verbal + Eyes) 2 = NA	Lowest value prior to airway management recorded on scene. NA = Not available

**Table 3:** Final airway management variables

FINAL AIRWAY MANAGEMENT VARIABLES (data provided by provider performing the intervention)				
Number	Data variable name	Type of data	Data variable categories or values Choose only one option unless otherwise stated.	Definition of data variable
19	Use of checklist for airway management	Ordinal	1 = Written checklist available and used on scene 2 = Written checklist available, but not used 3 = No checklist available	Written checklist for airway management including rapid sequence induction (RSI) available in service and used on-scene (challenge and response system).
20	Oxygenation strategy for airway management	Ordinal	1 = Preoxygenation with non rebreathable face mask before airway attempt 2 = Preoxygenation with Bag-valve-mask (BVM) before airway attempt 3 = Apnoeic oxygenation during airway attempt 4 = No preoxygenation	Oxygenation strategies used before or during advanced airway management. Select all that apply.
21	Sequence of providers performing airway management	Nominal	☐☐ Emergency Medical Technician ☐☐ Paramedic ☐☐ Nurse (non-anaesthesia) ☐☐ Nurse (anaesthesia) ☐☐ Physician (General practitioner or other non-EP/ICU/Anaesthesiologist) ☐☐ Physician (Emergency Physician - EP) ☐☐ Physician (Intensivist - ICU) ☐☐ Physician (Anaesthesiologist) ☐☐ Unknown	Specify level of EMS provider in sequence, who performed each airway management attempt, numbered in order of attempt. Select all that apply. Specify number of attempt alongside corresponding provider with “1” and if more attempts “2”, “3”,“4”. E.g.: If paramedic fails first attempt, then physician has two attempts, this is recorded as: “1” Paramedic. “2–3” Physician. Select all that apply.
22	Sequence of airway devices used for airway management	Nominal	☐☐ Bag-valve-mask ventilation (BVM) ☐☐ Supraglottic airway device with suction ☐☐ Supraglottic airway device without suction ☐☐ Direct laryngoscopy with endotracheal tube ☐☐ Direct laryngoscopy with endotracheal tube and stylet ☐☐ Direct laryngoscopy with bougie and endotracheal tube ☐☐ Video laryngoscopy (Macintosh or Miller like blade) with endotracheal tube ☐☐ Video laryngoscopy (Macintosh or Miller like blade) with endotracheal tube and stylet ☐☐ Video laryngoscopy (Macintosh or Miller like blade) with bougie and endotracheal tube ☐☐ Video laryngoscopy (hyperangulated blade) with endotracheal tube ☐☐ Video laryngoscopy (hyperangulated blade) with endotracheal tube and stylet ☐☐ Video laryngoscopy (hyperangulated blade) with bougie and endotracheal tube ☐☐ Surgical emergency airway equipment ☐☐ Percutaneous emergency airway equipment ☐☐ Jet-ventilation equipment ☐☐ Other, describe: _______________ ☐☐ Unknown	Specify first attempt with “1” and if more attempts “2”, “3”,“4”. E.g.: If first attempt fails with endotracheal intubation and direct laryngoscopy, and the next two attempts are endotracheal intubation with video laryngoscopy, this is recorded as: “1” Direct laryngoscopy with endotracheal tube “2–3” Video laryngoscopy (Macintosh or Miller like blade) with endotracheal tube BVM = Bag-valve-mask ventilation, includes insertion of oro/nasopharyngeal airway. If bag-valve-mask ventilation prior to RSI, choose “BVM” as “1”. Video laryngoscopy (VL) differentiates between: VL with Macintosh/miller like blade VL with hyperangulated blade Select all that apply.
23	Airway management results	Ordinal	1 = Successful airway management with ET as planned 2 = Successful airway management with SAD as planned 3 = Successful airway management with surgical airway as planned 4 = Failure of primary airway plan, and airways secured by alternative technique 5 = Final airway management failed (loss of airways) 6 = Unknown	ET = Endotracheal tube SAD = Supraglottic airway device
24	Airway manoeuvres following failed airway attempt.	Nominal	1 = Cricoid pressure released 2 = BURP/ELM manoeuvres 3 = Release MILS 4 = Reposition patient 5 = Ramping patient 6 = None / Not applicable.	Airway manoeuvres following unsuccessful airway management attempts. BURP = Backwards upwards rightwards pressure. ELM = External laryngeal manipulation MILS = Manual In-line stabilisation Ramping = The head and trunk are elevated or supported to align the external auditory meatus with the sternal notch in the horizontal plane Select all that apply.
25	Drugs used to facilitate airway management	Nominal	1 = None 2 = Thiopental 3 = Ketamine 4 = S-ketamine 5 = Propofol 6 = Fentanyl 7 = Alfentanil 8 = Morphine 9 = Midazolam 10 = Diazepam 11 = Suxamethonium 12 = Rocuronium 13 = Vasopressor 14 = Lidocain 15 = Etomidate 16 = Other, describe: ________________	Drugs used to facilitate the actual airway intervention, not including sedation in the post-intervention or transport phase. Vasopressor includes any drug used as vasopressor during airway management, e.g. phenylephrine. Local anaesthetic includes any drug used as local or regional anaesthetic, e.g. lidocaine. Select all that apply.
26	Complications during airway management	Nominal	1 = ET misplaced in oesophagus AND recognised/corrected immediately 2 = ET misplaced in oesophagus and NOT recognised/corrected immediately 3 = ET misplaced in left or right main stem bronchus 4 = Incorrect positioning or difficult ventilation with SAD 5 = Dental trauma 6 = Aspiration or vomiting during airway management (and NOT present before) 7 = Cardiac arrest during airway management 8 = Hypoxia during airway management 9 = Bradycardia during airway management 10 = Hypotension during airway management 11 = Complications during surgical or percutaneous airway (e.g. bleeding or pneumothorax) 12 = No complications (confirmed) during airway management 13 = Insufficient data recording, complications unsure.	Complications recognised during airway management or device verification (and NOT present before the airway management). Select all that apply. ET = Endotracheal tube, SAD = Supraglottic airway device The following definitions are used:Hypoxia: *Adults and children: SpO2 < 90%* Hypotension: *infants < 1 year: SBP < 70 mmHg* *children 1 to 10 years: SBP < 70 + (2 × age)* *children > 10 years: SBP < 90 mmHg* *adults: SBP < 90 mmHg or decrease > 10% from baseline value* Bradycardia *newborn to 3 years: < 100 bpm* *3 to 9 years: < 80 bpm* *10 to 16 years: < 60 bpm* *adults: < 50 bpm* Select all that apply.
27	Total number of successful endotracheal intubations the provider has performed in patients	Ordinal	0 = < 10 1 = 11–25 2 = 26–50 3 = 51–100 4 = 101–250 5 = 251–1000 6 = 1001–2500 7 = > 2500	Total number of successful endotracheal intubations the provider has performed in patients in hospital and pre-hospital service, not including mannequin intubations or SAD.
28	Blood pressure, after finalised airway management	Continuous and Nominal	1 = Number, describe (Syst-BP/Dias-BP (MAP)) 2 = NA: Did not measure 3 = NA: Could not measure	Value recorded within 1–3 min after finalised airway management
29	SpO_2_, after finalised airway management	Continuous and Nominal	1 = Number, describe 2 = NA: Did not measure 3 = NA: Could not measure	Value recorded within 1–3 min after finalised airway management
30	EtCO_2_, after finalised airway management	Continuous and Nominal	1 = Number, describe 2 = NA: Did not measure 3 = NA: Could not measure	Value recorded within 1–3 min after finalised airway management
31	Ventilation, after finalised airway management	Nominal	1 = Spontaneous ventilation 2 = Controlled manual ventilation 3 = Controlled mechanical ventilation (ventilator) 4 = Mixed ventilation 5 = Unknown	Main mode of ventilation on-scene and during transport of patient following finalised airway management. If both spontaneous and controlled ventilation, choose “mixed ventilation”
32	Survival to hospital	Nominal	1 = Dead on-scene after ALS interventions 2 = Alive on hospital arrival (including patients being transported with on-going mechanical chest compressions or ECPR) 3 = Unknown	Patient survival status limited to pre-hospital treatment and arrival at hospital (Short term survival) ALS = Advanced Life Support ECPR = extracorporeal cardiopulmonary resuscitation

**Table 4:** Final system variables

FINAL SYSTEM VARIABLES (data provided by Medical Director EMS)				
Number	Data variable name	Type of data	Data variable categories or values Choose only one option unless otherwise stated.	Definition of data variable
33	Established airway management procedure (SOP)	Ordinal	1 = Yes, SOP with Checklist 2 = Yes, SOP only 3 = No SOP	SOP including algorithm for difficult intubation (expected/unexpected) available in EMS service.
34	Type of airway currency training in service	Nominal	1 = Clinical rotation with regular airway management practise (e.g. anaesthesia) 2 = Regular airway management currency assessments (e.g. RSI simulation) 3 = Regular mannequin training 4 = Regular cadaver training 5 = Other, describe	Clinical rotation: describes system with regular airway management currency (e.g. anaesthesia practise). Regular airway management currency assessment (e.g. RSI simulation) describes systems with simulation or virtual training for airway management currency. Cadaver and mannequin describes systems with regular airway management skill training. Select all that apply.
35	Type of tracheal tube confirmation technique used in service	Nominal	1 = Auscultation only 2 = Capnometry only 3 = Waveform capnography 4 = Colorimetric detector (e.g. Easycap) 5 = Ultrasound 6 = Other, describe: _______________ 7 = None	Capnometry is a measurement of ETCO_2_ i.e., analysis alone) without a continuous written record or waveform. Waveform capnography includes waveforms of inspiration and expiration pattern along with values for ETCO_2_. Select all that apply.
36	Airway management devices used in service	Nominal	1 = Bag-valve-mask ventilation 2 = Supraglottic airway device with suction 3 = Supraglottic airway device without suction 4 = Direct laryngoscopy with endotracheal tube (including bougie and/or stylet). 5 = Video laryngoscopy with Macintosh or Miller like blade and endotracheal tube (including bougie and/or stylet). 6 = Video laryngoscopy with hyperangulated blade and endotracheal tube (including bougie and/or stylet). 7 = Surgical emergency airway equipment 8 = Percutaneous emergency airway equipment 9 = Jet-ventilation equipment 10 = Other, describe: _______________ 11 = Unknown	Airway devices available in service and provider who knows how to use it. Bag-valve-mask ventilation includes insertion of oro/nasopharyngeal airway. Video laryngoscopy (VL) differentiates between: VL with Macintosh/miller like blade VL with hyperangulated blade Select all that apply.
37	Drugs for airway management available in service	Nominal	1 = None 2 = Thiopental 3 = Ketamine 4 = S-ketamine 5 = Propofol 6 = Fentanyl 7 = Alfentanil 8 = Morphine 9 = Midazolam 10 = Diazepam 11 = Suxamethonium 12 = Rocuronium 13 = Vasopressor 14 = Lidocain 15 = Etomidate 16 = Other, describe: ______________	Drugs used for airway management, available on scene and someone competent to administer them. Select all that apply.
38	Highest Level of EMS provider involved in airway management on-scene	Nominal	1 = Emergency Medical Technician (EMT) 2 = Paramedic 3 = Nurse (non-anaesthesia) 4 = Nurse (anaesthesia) 5 = Physician (General practitioner or other non-EP/ICU/Anaesthesiologist) 6 = Physician (Emergency Physician - EP) 7 = Physician (Intensivist - ICU) 8 = Physician (Anaesthesiologist) 9 = Unknown	Highest level of EMS provider present on scene and involved in airway management; including assessment, drugs or intervention.

## Discussion

### Main results

Using a modified nominal group process with international airway experts, supported by clinical study results with the original template described in the recent AIRPORT studies, we have revised the template for the reporting of standardised data from PHAAM [22, 23]. The updated dataset includes new data points that reflect risk factors for difficult PHAAM, the use of checklists and standard operating procedures (SOPs), strategies for pre-oxygenation, the use of drugs in PHAAM, airway currency training, developments in airway devices, airway management strategies, and patient safety issues not previously described in the Utstein-style airway template.

### Time variables

Three time intervals are important for describing a pre-hospital response adequately: the response time, on-scene time, and patient transport time to the hospital. In comparing EMS or dispatch services across patient populations and services, these intervals are valuable for describing the EMS response, which is also closely linked to the efficiency of the dispatch process [24].

### Patient variables

Patient age and sex should be included in any study population demographics [5]. We have previously shown a non-linear association between patient age and the first-attempt TI failure rates and that a significant age difference exists between trauma and non-trauma patients intubated by physician-staffed helicopter emergency medical services (HEMS), indicating that it is important to include age in a PHAAM dataset [22]. A sex difference has also previously been described in emergency airway management [25].

Category describes the dominating reason for the emergency treatment, while indication describes the dominating indication for the airway intervention itself. While the patient category is among the variables most consistently reported (86%) in airway studies, indication is less frequently reported (36%) [5]. Describing trauma cases, differentiating between blunt trauma and penetrating trauma may be important, as strategies for both airway management and haemorrhage control can differ between these groups [26]. Traumatic brain injury (TBI) is another major category where treatment options may differ, and airway management competence is linked to mortality in this group [27]. In non-trauma patients, the experts agreed that distinguishing between cardiac arrest; neurological emergencies; respiratory distress or breathing difficulties; intoxication; infection (including sepsis); and other medical emergencies may be important when describing PHAAM.

Airway assessment is an integral part of providing safe pre-hospital anaesthesia and advanced airway management. Patient risk factors for difficult bag-valve-mask (BVM) ventilation or TI were not included in the original template. Optimal patient positioning may maximise the chance of successful PHAAM [20]. And, the pre-hospital setting contains some unique external factors, which may influence access to the patient and hence airway management success [28, 29]. The expert panel agreed that such risk factors should be described in the dataset.

Key vital signs are commonly used to assess the physiological status of patients in many clinical settings [30]. The experts agreed that single values, not ranges of measurements, should be recorded in general. Agreeing on the necessity of an initial baseline measurement of the patient’s respiratory rate (RR), blood pressure, peripheral oxygen saturation (S_P_O_2_) and Glasgow coma score (GCS), the experts also found that recording the lowest value prior to and during the airway intervention was important. The recording of end-tidal carbon dioxide (ETCO_2_) after finalised PHAAM is important to confirm tube placement but may also be beneficial for optimising advanced life support (ALS) [31].

### Airway management variables

The use of pre-TI checklists for PHAAM to reduce adverse events and improve patient safety is recommended [32]. However, a recent multicentre randomised trial of checklist use in rapid sequence intubation (RSI) found no reduction in complication rates compared to standard practice without checklists [33]. While experienced providers may rely on mental checklists, inexperienced providers may depend on written checklists in a challenge and response system. The experts agreed that the airway dataset should only document whether a written checklist is available on scene and whether the checklist was used.

The Difficult Airway Society recommends that pre-induction airway plans are briefed to the team and that failure of primary or secondary airway plans are clearly declared to facilitate control of the patients’ airways and to avoid complications [20]. Although a prediction of a difficult airway is not always reliable, a planned and verbalised pre-induction airway plan should be in place prior to an RSI [20, 34]. The experts agreed that recording whether PHAAM was successful as planned and documenting whether the final airway attempt failed were important.

There is evidence for an association between airway management skills and patient outcomes in PHAAM [27, 35]. Poorly performed airway management carries significant mortality and morbidity risks, and adequate training and experience is important for patient safety [35]. As success and complication rates are also associated with provider competence and experience, the experts found that recording the level and sequence of providers performing the actual airway interventions was necessary [36]. Specifying the sequence of providers may provide new knowledge of PHAAM, especially where primary airway management fails and an unanticipated difficult airway in the field must be handled. Furthermore, the results from airway studies are difficult to interpret and compare without such information. The total number of successful TIs the provider has performed in patients in hospital and in pre-hospital service may be regarded as a reasonable surrogate for total airway competence [37].

Patient pre-oxygenation is standard practice during any anaesthesia induction, aiming at maximising the oxygen reserves and delaying the onset of desaturation for several minutes in the event of a failed or difficult primary airway intervention [38]. Strategies for pre-oxygenation have improved over the last decades, targeting both the pre-induction phase, and the apnoea time after the induction of anaesthesia [39]. Pre-oxygenation was not included in the original template; however, the experts agreed that as pre-oxygenation might have a crucial effect on avoiding hypoxia during the apnoeic phase of TI and should be recorded [38].

Although RSI is standard practice for emergency anaesthesia in patients with a risk of pulmonary aspiration, the definition of RSI may not be uniform across international EMS services [32, 40]. RSI implies a transition from full consciousness with intact airway reflexes to complete unconsciousness. Ensuring optimal TI conditions with a high first pass success rate, backed up by rehearsed airway plans should the primary TI attempts fail, is important [20, 34]. Although the use of NMBA may increase TI success rates, a setting where the patient is rendered apnoeic may be challenging if primary airway management fails [36].

The most critical part of PHAAM may be the airway intervention itself, especially when performed during suboptimal conditions in the field [41]. Limiting the number of attempts is recommended, before declaring failed TI and proceeding with an alternative airway device [20]. Thus, specifying the number of attempts and type of device used in each attempt in sequential order may be important when documenting airway complications as an integral part of a patient safety culture. Rescue manoeuvres, such as backwards-upwards-rightwards-pressure or external laryngeal manipulation are manoeuvres commonly used to optimise TI conditions [34].This was not included in the original template, but the experts found that describing these manoeuvres in the revised dataset was useful.

In recent years, video laryngoscopy (VL) has been increasingly used in airway management [42, 43]. Although VL may improve the glottic view and be beneficial in the context of a difficult airway, little evidence exists today showing that VL reduces the number of TI attempts or airway complication rates, compared to direct laryngoscopy (DL) [44, 45]. As a technique involving an airway device, VL was not included in the original template. The benefit of VL in PHAAM still needs to be demonstrated, therefore the main types of VL and DL were included in the revised dataset.

The possibility of isolating different generic drugs used for PHAAM across patient categories might provide new knowledge, and the experts agreed to include the most common generic drugs used in PHAAM today.

The experts agreed that survival to hospital (short-term survival) should be recorded. Additionally, “dead on arrival” implies that no ALS procedures have been provided and should not be included. Rather, “dead on-scene after ALS interventions” or “alive on hospital arrival” should be documented. This categorisation includes patients being transported to the hospital with on-going mechanical chest compressions or extracorporeal cardiopulmonary resuscitation (ECPR) [46].

### System variables

SOPs, including algorithms for unexpected difficult airway management, are emerging as an indispensable part of patient safety and quality systems [32]. The experts agreed that recording whether airway management SOPs are available in the individual EMS is important, also recognising the importance of developing robust clinical governance systems for pre-hospital critical care [47]. The experts agreed that the types of recurring airway management training provided by the EMS systems are important to record.

### Value of standardised data

To be able to compare interventions or level of care across systems, standardized research data using common terminology, data definitions or quality indicators are required [48]. Developing common variables and definitions is an on-going process and it is important to identify the correct variables to use in airway research projects and for benchmarking of airway management across EMS [14]. The results from consensus processes such as ours are not the endpoint, as dissemination and implementation of the results into clinical service are the final aims [49]. A few EMS have successfully implemented the original template into clinical service; however, endorsement by internationally recognised airway societies, research groups, or key EMS stakeholders, along with interoperable health information systems, may be vital to secure a broad implantation of the airway template in international EMS [8, 50, 51].

The feasibility of collecting airway and trauma data using standardised templates have been shown, and it is important that data in comparative research projects are collected in a uniform manner [22, 48, 52]. To date, twenty-two articles have described data collection methods adhering to, or adapted to, the original Utstein-style airway template [3, 5, 8, 21–23, 50, 52–66]. A further twenty-three articles have referred to the original publication [6, 8, 13, 16, 35, 36, 41, 67–82]. However, as technology evolves, the availability of new possibilities of data capture from devices like video or body cameras, or streaming of monitor data directly to hospital data systems, may influence this kind of research [83, 84]. Therefore, how study data are obtained may be important to document to increase accuracy of data.

### Limitations

The scientific value of consensus methods, such as mNGT or the Delphi surveys, have been questioned, and no method is considered a “gold standard” [85]. Nonetheless, consensus methods are useful tools to assess agreement on questions for which hard evidence is difficult to obtain. We believe that recruiting a broad panel of experts according to predefined criteria, from fourteen countries across Europe, Australia and United States of America, may have reduced a possible selection bias and yielded a representative list of variables with scientific value. Keeping the preliminary email rounds and proposals anonymous from round one to three was important to avoid the influence of “loud-speaking” experts and to reduce the effect of a strong reputation or opinion on other more “silent-speaking” experts [9]. Each QA round was handled confidentially so that the experts were not aware of the answers or comments from the other experts.

## Conclusions

Using a mNGT with international experts, we have updated the dataset to report standardised data from pre-hospital advanced airway management. The dataset enables future airway management research to produce comparable high-quality data across emergency medical systems. We believe this approach will promote research and improve treatment strategies and outcomes for patients receiving pre-hospital advanced airway management.’

## Additional files

Additional file 1: Questionnaire first email round. (XLSX 24 kb)

Additional file 2: Questionnaire second email round. (XLSX 21 kb)

Additional file 3: Questionnaire third email round. (XLSX 20 kb)

Additional file 4: Expert group composition. (DOCX 140 kb)

Additional file 5: Questionnaire consensus meeting. (XLSX 17 kb)

## Abbreviations

ALS: Advanced life support
BVM: Bag-valve-mask
DL: Direct laryngoscopy
ECPR: Extracorporeal cardiopulmonary resuscitation
eFONA: Emergency front of neck access
EMS: Emergency medical services
ETCO_2_: End-tidal carbon dioxide
GCS: Glasgow coma score
HEMS: Helicopter emergency medical services
mNGT: Modified nominal group technique
NMBA: Neuromuscular blocking agents
PHAAM: Pre-hospital advanced airway management
QA: Questionnaires and answers
RR: Respiratory rate
RSI: Rapid sequence intubation
SAD: Supraglottic airway device
SOP: Standard operating procedure
S_P_O_2_: Peripheral oxygen saturation
TBI: Traumatic brain injury
TI: Tracheal intubation
VL: Video laryngoscopy
