# Supplementary material for: Standardised data reporting from pre-hospital advanced airway management – a nominal group technique update of the Utstein-style airway template
Source: Scand J Trauma Resusc Emerg Med. 2018 Jun 4;26:46. doi: 10.1186/s13049-018-0509-y (PMC5987657; doi:10.1186/s13049-018-0509-y)
Supplement: Supplementary file 4 — Expert group composition. (DOCX 140 kb) [file 13049_2018_509_MOESM4_ESM.docx]

**Supplement-1 - Expert group composition.**

| **Expert** | **Airway management background and experience** |
| --- | --- |
| Ilkka Virkkunen  **(Finland)** | Ilkka Virkkunen has worked 17 years as a HEMS physician (consultant in Anaesthesiology and Intensive Care since 2004) at three different HEMS bases and is now leading the FinnHEMS R&D Unit. He has also worked as a medical director in emergency medical services at Tampere University Hospital for six years. He made his thesis on out of hospital cardiac arrest patients. |
| Wolfgang Voelckel  **(Austria)** | Wolfgang Voelckel, M.D. Anaesthesiologist, board certified in 1994. Involved in teaching and simulation since 1996 with a special interest in airway, trauma care and CPR. Co-investigator and co-author of prospective, randomized pre-hospital airway trials in ÖAMTC Austrian Air Rescue (AirTraq^TM^, Glidescope^TM^ and Video-laryngoscopy). Organizer of recurrent airway workshops (i.e. http://www.intensivkompakt.at).​  Medical director ÖAMTC Austrian Air Rescue, responsible for medical quality management and system development (i.e. introduction of video-laryngoscopy). |
| Peter Paal  **(Austria)** | Peter Paal MD PD MBA EDAIC EDIC, consultant in anaesthesia and intensive care medicine, head of department at the Hospitallers Brothers Hospital Salzburg, Austria. For several years deputy lead of the maxillofacial surgery anaesthesia team Innsbruck University Hospital and paediatric anaesthesia team member, HEMS Christophorus Austria. Scientific co-lead of the airway management group of the Austrian Society of Anaesthesiology, Resuscitation and Intensive Care Medicine (OEGARI). Lead organiser of the Innsbruck Airway Symposium for several years. 30+ publications in peer reviewed journals on airway management. |
| Lorenz Theiler  **(Switzerland)** | PD Dr med Lorenz Theiler works as senior attending anesthesiologist at the University Hospital Inselspital in Bern, Switzerland. He is head of the division of emergency anesthesia, prehospital anesthesia and ambulatory anesthesia and he also works as research officer for the Swiss air rescue organisation Rega. His main research interest covers all aspects of airway management and he published numerous papers in this area. Lorenz Theiler is co-secretary and member of the board of directors of the European Airway Management Society EAMS and editor of the airway section of the journal Trends in Anaesthesia and Critical Care TACC. He organizes the pre-congress course in airway management at the annual meeting of the European Society of Anaesthesiology ESA as well as the airway courses at his teaching hospital. |
| Massimiliano Sorbello  **(Italy)** | Massimiliano Sorbello, Anesthesiologist. Long time research, scientific activity, education and clinical practice in the field of airway management. Actually, Chairing Airway Management research group in SIAARTI (Società Italiana Anestesia Analgesia Rianimazione e Terapia Intensiva), including the field of research for prehospital airway management. Coworker for production, diffusion and educational of SIAARTI-PAMIA (Prehospital airway management italian association :http://www.siaarti.it/Ricerca/Linee-guida-per-la-gestione-preospedaliera-delle-vie-aeree.aspx). Actually, Chairing European Society of Anaesthesiologists - Subcommittee 11 Respiration and Airway Management, which includes field of research for prehospital airway management. Tutor-teacher in many airway courses, providing also simulation, including the field of prehospital airway management. Based in Catania University Hospital, in Italy, Massimiliano Sorbello is co-author of SIAARTI Guidelines for airway management in adult and paediatric patient, for slung separation procedures and author or co-author of many scientific papers and book chapters in the field of airway management. SIAARTI member (Research committee chair), ESA member (SC11 chair) and European Airway Management Society Board member. |
| Michael Gellerfors  **(Sweden)** | Mikael Gellerfors is Research Director at the Swedish Air Ambulance (SLA) and Medical Director at the Ambulance Helicopter in Dalarna. Mikael works as Senior Consultant at Dalarna Ambulance Helicopter. Additionally, he serves as Critical Care Physician with the Rapid Response Car in Stockholm and anaesthesiologist at Södersjukhuset hospital. Mikaels research centers on prehospital advanced airway management. Mikael is board member of the Swedish Association of Physicians in Prehospital Emergency Medicine and member of the Nordic Prehospital Research Alliance working group. |
| Richard Lyon  **(England)** | Richard Lyon is an Associate Medical Director and Research Lead for Kent, Surrey & Sussex Air Ambulance and Professor of Pre-hospital Emergency Care at the University of Surrey in the UK. I have led on several key HEMS research papers, including on a new pre-hospital drug regimen for RSI and am involved in Standard Operating Procedure writing, Clinical Governance and training of pre-hospital airway management for both trauma and medical cases. |
| Kate Crewdson  **(England)** | Kate Crewdson is a consultant in Anaesthetics and Intensive Care Medicine in Bristol UK. I have worked as a Pre-hospital Emergency Physician with London’s Air Ambulance, UK. I am currently undertaking a higher research degree, MD (Res) in Prehospital Advanced Airway Management. |
| Brian Burns  **(Australia)** | Brian Burns is a consultant with Greater Sydney Area Helicopter Emergency Medical Service, New South Wales Ambulance, Sydney, NSW, Australia; and with Discipline of Emergency Medicine, Sydney Medical School, Sydney, NSW, Australia. His research focus is on prehospital retrieval medicine and airway management. |
| Leif Rognås  (**Denmark)** | Leif Rognås is a consultant anaesthesist at Aarhus University Hospital in Denmark, Lead Clinician for the pre-hospital critical care service i Aarhus and HEMS consultant with the Danish Air Ambulance. He is an associate professor of pre-hospital care at Aarhus University and holds a PhD on pre-hospital advanced airway management. In 2016, Leif co-authored the Scandinavian guideline for pre-hospital airway management. |
| Mårten Sandberg  **(Norway)** | Mårten Sanderg is a specialist of anesthesiology, professor in prehospital emergency medicine at University of Oslo, Norway, and senior consultant in the Air Ambulance Department at Oslo University Hospital, Oslo, Norway. He has done research in various aspects of prehospital airway management and is a co-author of the Scandinavian SSAI clinical practice guideline on pre-hospital airway management (2008, 2016). |
| Björn Hossfeld  **(Germany)** | Bjoern Hossfeld works as senior consultant in the Dept. of Anaesthesiology & Intensive Care Medicine at German Armed Forces Hospital Ulm. He is speaker of the Section Emergency Medicine of the German Interdisciplinary Association for Intensive Care and Emergency Medicine. His research focus is on difficult airway management and especially on video laryngoscopy out-of-hospital. |
| Alasdair Corfield  **(Scotland)** | Alasdair Corfield is a consultant in Emergency, Prehospital & Retrieval Medicine based with EMRS/ScotSTAR in Glasgow, Scotland, where he is research lead. He is an honorary associate professor at the University of Glasgow, and has previously researched and published on many aspects of prehospital care, including advanced airway management. |
| Daniel Davis  **(USA)** | Daniel Davis, MD, is medical director for both ground and air EMS programs and is responsible for resuscitation training for Air Methods Corporation, one of the largest air medical services in the world. He has performed extensive research in prehospital airway management and developed the Advanced Resuscitation Training (ART) program, with out-of-hospital intubation success rates in excess of 99 percent among flight nurses and paramedics. |
| Tomasz Gaszyński  **(Poland)** | Tomasz Gaszynski is a specialist in anaesthesiology and intensive therapy. professor of Anaesthesia, Head of Department of Anaesthesiology and Intensive Therapy, Medical University of Lodz, Poland, formerly Head of Department of Emergency and Disaster Medicine Medical University of Lodz, Poland. Co-founder and President of of Polish Airway Management Society (branch of Polish Society of Anesthesiology and Intensive Therapy), member of Scientific Committee European Airway Management Society and member of Council of European Airway Management Society. Researcher and author of many scientific publications on airway management including pre-hospital and emergency airway management. One of the author of Polish Guidelines for Difficult Airway Management and Polish Guidelines for Pre-hospital Airway Management and Polish Guidelines for Paediatric Difficult Airway Management. |
| Pavel Michalek  **(The Czech Republic)** | Prof. Pavel Michalek, MD PhD DESA MSc, is a consultant in Anaesthesia and Intensive Care Medicíne. Location: Dept of Anaesthesia and Intensive Care, General University Hospital in Prague, Czech Republic. Dept of Anaesthesia and Intensive Care, Antrim Area Hospital, Antrim, UK. Instructor for advanced airway training - anesthetists, paramedics. Working as a Locum Physician for the Czech Ambulance Services between 1995-2005. |
